# A Minimally Invasive Microsensor Specially Designed for Simultaneous Dissolved Oxygen and pH Biofilm Profiling

**DOI:** 10.3390/s19214747

**Published:** 2019-11-01

**Authors:** Xavier Guimerà, Ana Moya, Antonio David Dorado, Xavi Illa, Rosa Villa, David Gabriel, Xavier Gamisans, Gemma Gabriel

**Affiliations:** 1 Department of Mining Industrial and ICT Engineering, Universitat Politècnica de Catalunya, Avinguda de les Bases de Manresa 61-73, 08242 Manresa, Spain; 2 Instituto de Microelectrónica de Barcelona IMB-CNM (CSIC), Esfera UAB, Campus Universitat Autònoma de Barcelona, 08193 Bellaterra, Spain; 3 CIBER de Bioingeniería, Biomateriales y Nanomedicina (CIBER-BBN), Spain; 4 GENOCOV Research Group, Department of Chemical, Biological and Environmental Engineering, Universitat Autònoma de Barcelona, 08193 Bellaterra, Spain

**Keywords:** DO microsensor, pH microsensor, multi-analyte sensor, MEMS technology, biofilm profiling, biofilm monitoring

## Abstract

A novel sensing device for simultaneous dissolved oxygen (DO) and pH monitoring specially designed for biofilm profiling is presented in this work. This device enabled the recording of instantaneous DO and pH dynamic profiles within biofilms, improving the tools available for the study and the characterization of biological systems. The microsensor consisted of two parallel arrays of microelectrodes. Microelectrodes used for DO sensing were bare gold electrodes, while microelectrodes used for pH sensing were platinum-based electrodes modified using electrodeposited iridium oxide. The device was fabricated with a polyimide (Kapton^®^) film of 127 µm as a substrate for minimizing the damage caused on the biofilm structure during its insertion. The electrodes were covered with a Nafion^®^ layer to increase sensor stability and repeatability and to avoid electrode surface fouling. DO microelectrodes showed a linear response in the range 0–8 mg L^−1^, a detection limit of 0.05 mg L^−1^, and a sensitivity of 2.06 nA L mg^−1^. pH electrodes showed a linear super-Nernstian response (74.2 ± 0.7 mV/pH unit) in a wide pH range (pH 4−9). The multi-analyte sensor array was validated in a flat plate bioreactor where simultaneous and instantaneous pH and DO profiles within a sulfide oxidizing biofilm were recorded. The electrodes spatial resolution, the monitoring sensitivity, and the minimally invasive features exhibited by the proposed microsensor improved biofilm monitoring performance, enabling the quantification of mass transfer resistances and the assessment of biological activity.

## 1. Introduction

Air pollution control has become an essential issue to ensure the health and the welfare of future societies as well as to limit and reduce the degradation of the environment [1]. For this reason, several treatment technologies have been developed and implanted to reduce the emission of gaseous pollutants and odors [2,3]. In addition, existing treatment technologies have been optimized to increase both their performance and operating ranges.

In this sense, biofilm-based technologies have shown competitive in front of physical-chemical technologies due to their higher energetic and economic efficiency and because they are more environmentally sustainable [4,5]. Bioreactors, and mainly biofilters and biotrickling filters, have been implanted for the treatment of a wide range of pollutants and conditions through the study of the phenomenon taking place within the liquid and gas phases [6,7]. The improvement of reactor monitoring has improved the knowledge of the mass transport and degradation of pollutants as well as the optimization of bioreactor performance. However, the lack of information about biofilm dynamics and the difficulties in monitoring within biofilms indicate that the future improvement of biofiltration technologies is linked to the development of monitoring tools.

The technical limitations for the study of biofilms by in situ measurements (due to their reduced thickness ranging from hundreds of microns to a few millimeters) have been partially solved by the development of several microsensors, both electrochemical [8,9] and optical [10,11]. Microsensors specific for the detection of different chemical species have been developed, enabling their monitoring inside biofilms with a high spatial resolution and with a low impact on biological systems. However, the recording of concentration profiles within biofilms using commercial microsensors requires the use of positioning devices with high precision to place microsensors mechanically through biofilms [12]. Often, only one chemical compound can be monitored within the biofilm [8]. In addition, devices are costly and very fragile [13].

Microelectromechanical systems (MEMS) technology has shown a high potential for the development of microsensors [14,15,16]. In this sense, this technology has been successfully used for the design and construction of electrochemical microsensors for the detection of several species, mainly dissolved oxygen (DO) [15,16,17], pH [14,18], and redox potential [19]. Microsensors based on MEMS technology reduced some of the limitations presented by conventional microsensors, both electrochemical [20] and optical [10]. The flexibility in design and mass production offered by MEMS technology has simplified the procedure for concentration profile recording and has reduced the cost. Improvements in the monitoring of biofilms using MEMS microsensors has resulted in the acquisition of relevant information of the phenomena involved [21]. However, MEMS microsensors still present some limitations such as a low stability of their response due to sensors configuration (naked electrodes) and the invasive nature of measurements due to device size. Consequently, the potential of this technology must be further investigated to improve the performance of the developed sensors and broaden their application.

On the other hand, MEMS technology can be used for the design of multi-electrode devices, giving rise to multi-analyte microsensors [22,23]. Such devices have been used mainly for monitoring microfluidic systems [24,25,26,27]. The development of these devices for the monitoring of specific biofilms will improve the study of systems in which the simultaneous monitoring of several analytes is required (such as nitrifying and sulfuroxidant biofilms).

With this aim, a previous microsensor prototype specially designed for DO profiling in biofilms [15] has been modified integrating several technological improvements. Thereby, the thickness of the novel device was minimized using a thin polymer layer as a needle substrate, and the electrode stability was increased including a protective membrane preventing one of their major drawbacks, which is the fouling of the electrodes arising from the deposition of unwanted organic material over their surface. The novel prototype consisted of two parallel multielectrode arrays of seven electrodes, each one for the simultaneous monitoring of DO and pH. The sensor response was characterized to test its performance for DO and pH monitoring. Finally, the prototype was experimentally validated comparing its behavior against a commercial DO Clark-type microsensor and a commercial glass-type pH microsensor during the profiling of the activity of sulfide oxidizing bacteria (SOB) grown in a laboratory-scale reactor.

## 2. Materials and Methods

### 2.1. Design and Fabrication of a Multi-Analyte Microsensor

**Figure 1:**
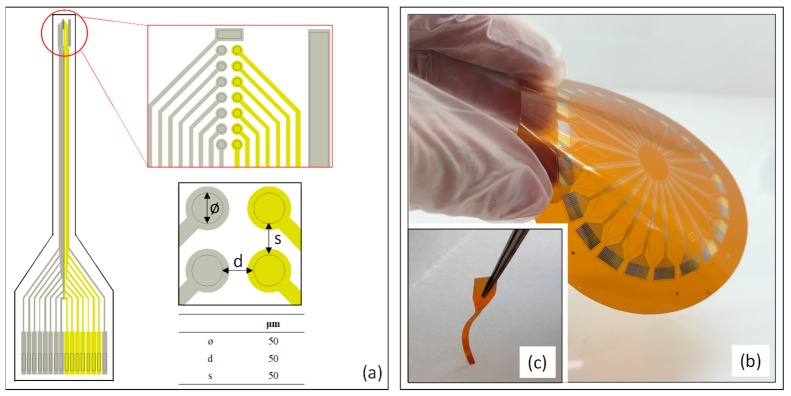
(**a**) Microsensor design diagram, consisting of 2 arrays of 7 disk electrodes fabricated in gold and in platinum, and an integrated pRE and CE. Details of the arrangement of the two arrays and corresponding diameter of the electrodes, the distance between them, and the distance between the two arrays. (**b**) A flexible polyimide multi-analyte sensor fabricated on a flexible Kapton^®^ substrate at a wafer level. (**c**) An individualized multi-analyte sensor.

The novel microsensor included, in a single needle, two parallel arrays of seven disk electrodes, each one separated by 50 µm (Figure 1). The disk electrodes of 50 μm in diameter were arranged equidistantly, separated also by 50 μm. This configuration allows the instantaneous acquisition of a DO and a pH profile along the biofilm depth by monitoring simultaneously both arrays. DO monitoring was based on amperometric oxygen detection and pH monitoring was based on potentiometric pH detection through solid-state electrodes. Several metal-oxide-based pH sensors have been developed, but IrOx electrodes were selected since they showed a high potential stability over a wide range of temperatures and pressures in an aqueous solution [28], a fast response even in non-aqueous solutions [29], the lowest sensitivity to redox interferences [30], and an excellent biocompatibility [31]. The array for DO detection was fabricated in gold (Au), while the array for pH detection was fabricated in platinum (Pt), since it offers a higher adherence for electrodeposited IrOx films [18,31].

The small dimensions of biofilms grown in packed-bed reactors prevent the use of external reference (RE) and auxiliary (CE) electrodes required for the operation of the microsensor. For this reason, the new device was specifically designed by integrating in the needle a reference system consisting in a rectangular platinum electrode (150 μm × 50 μm), designed as a pseudo-reference electrode (pRE), and a rectangular platinum electrode (2400 μm × 114 μm).

The multi-analyte microsensor was fabricated in the clean room facilities at the Institute of Microelectronics of Barcelona (IMB-CNM, CSIC, Bellaterra, Spain) at a wafer level [32]. In brief, 10/100 nm Ti/Au and Ti/Pt layers were evaporated on the 127-µm-thick polyimide film (Kapton^®^ 500HN, DuPont, Midland MI, USA) to fabricate the electrodes. The Ti layer was used to improve the adhesion between Au and Pt electrodes and the polymeric substrate. The active area of the electrodes and the pad connection were defined using the negative photoresist SU-8 material due to its optimal dielectric properties. The needles were individualized by cutting them using a cutter plot (Roland GX-24, Irvine CA, USA) obtaining 22 sensors per wafer. Finally, the connection was done using a zero insertion force (ZIF) connection system, reducing the time and cost of manufacturing.

### 2.2. Electrode Preparation

Microelectrode activation before their use was required. To this aim, several metal-cleaning methods were investigated [33]. The cleaning protocol selected for microelectrode activation consisted of needle washing for 10 min in a solution of 50 mM KOH and 25% H_2_O_2_ before rinsing with Milli-Q water. In particular, three consecutive washings were established in Moya et al. [15] to ensure optimal electrode activation. Adequate electrode activation was assessed by Ip and ΔEp values consistent with reversible kinetics. For this, dynamic cyclic voltammetry (CV) measurements in ferro/ferricyanide (10 mM) were performed. Microelectrodes were cleaned in ethanol for 5 min and rinsed in Milli-Q water to remove grease and organic deposits before their activation.

Pt electrodes of the fabricated needle was modified in order to perform pH measurements. After their activation, Pt disk electrodes were coated with a thin IrOx (K_1.7_ Ir_1.1_ (OH)_2.7_·0.5 H_2_O) film [31]. Electrodeposition of iridium salts through a dynamic sweep potential method [34] was selected since this procedure is compatible with the use of plastic substrates. This method allows for the obtainment of 300-nm-thick films over platinum electrodes. The solution used for the electrodeposition of IrOx films consisted of 1 mM IrCl_3_·H_2_O, 1 mM H_2_C_2_O_4_·2H_2_O, and 5 mM K_2_CO_3_. The final solution was kept at 37 °C for 4 days and stored at 4 °C. The IrOx electrodeposition was performed in a 3-electrode cell using an external CE (MC3051Pt, Radiometer Analytical, Villeurbanne, France), an external RE (REF321, Radiometer Analytical, Villeurbanne, France), and the 7 Pt electrodes as working electrodes (WE). The monitoring procedure was controlled through an 8-channel potentiostat (1010C, CH-Instruments, Austin TX, USA). The IrOx films were obtained applying a potentiometric protocol [18] consisting of 50 potential linear sweeps between open-circuit potential (OCP) (0.00 V) and 0.65 V (vs. Ag/AgCl), with a scan rate of 10 mV·s^−1^. During the procedure, WE and external CE were arranged in parallel to ensure a homogeneous coating of all the electrode. Modified needles were stored in a KCl (3 M) solution to avoid electromigration problems of potassium cations in the coating that could cause a drift in the response of the sensor.

### 2.3. Electrode Protection

The stability of microelectrodes over time was increased, isolating electrode surfaces from the measuring medium to avoid its passivation and fouling. The electrode protection was performed depositing a thin proton exchange membrane over the electrodes by immersing the needles in a Nafion^®^ solution (Nafion^®^ 117 solution, 5% in lower aliphatic alcohols, Sigma Aldrich, Saint Louis MO, USA) for 30 s. Needle position was controlled with the aid of a micromanipulator (MM3-2, Unisense, Aarhus, Denmark) to avoid the deposition of the membrane over the connectors. The protective film was consolidated keeping the needles in a horizontal position for 24 h to ensure the complete solvent evaporation. The needles were then hydrated in deionized water and stored in a saline solution (3 M KCl) to avoid Nafion^®^ dehydration, resulting in membrane cracking and deterioration. The thickness of the membranes was measured after their deposition using a profilometer (KLA-Tecnor profilometer, KLA-Tecnor, Milpitas CA, USA). The effect of the Nafion^®^ membrane on sensor performance was assessed during microsensor response characterization.

### 2.4. DO and pH Monitoring

The DO concentration was amperometrically monitored on the Au electrodes. For this purpose, the sensor was polarized against a reference system at the overall oxygen reduction potential. Several works [35,36] have suggested using an electrochemical potential between −800 and −900 mV (vs. Ag/AgCl). Nevertheless, a comprehensive assessment between oxygen reduction potential and DO sensitivity presented in Moya et al. [15] highlighted that optimal performance is obtained when −850 mV (vs. Ag/AgCl) is used. Although the needle design included an internal pRE, it was not possible to achieve a pRE with enough stable potential for the long-term measurements required in this work. For this reason, an external RE (CHI-111, CH-Instruments, Austi TX, USA) was finally used. The polarization of the electrodes and the recording of the oxygen reduction currents were carried out using a potentiostat (1010C, CH-Instruments, Austi TX, USA). The sensor was calibrated in the concentration range between 0 and 8 mg·L^−1^ of DO, adjusting the DO concentration in the calibration medium (0.1 M KNO_3_) by bubbling different nitrogen (0% O_2_)–air (21% O_2_) mixtures. The DO concentration in the calibration cell was measured with a commercial DO probe (Oxi 3310, WTW, Weilheim in Oberbayern, Germany) and was correlated with the measured polarization currents of each gold microelectrodes in order to build the calibration curves as described in Moya et al. [15]. On the other hand, pH monitoring on Pt electrodes coated with IrOx films was performed assessing the deviation from OCP as a function of the pH on the measuring medium. The simultaneous potentiometric measurement of pH on the 7 electrodes was performed using a multiplexed potentiostat (EmStatMUX16, Palmsens, Houten, The Netherlands) and the external RE. The calibration of the electrodes for pH detection was carried out, correlating the response of the microsensor, in terms of OCP, with the pH of 3 pH buffer solutions of pH 4.01, 7.00, and 9.21 (pH buffers, Crison, Alella, Spain). All measurements were performed under temperature and pressure conditions of 298 K and 1 atm, respectively.

Data from the calibration plots were used to quantify microsensor linearity and sensitivity for DO and pH detection. The calibration data were also used to evaluate the response of Nafion^®^ modified electrodes. The sensor characterization was completed, estimating quantification and detection limits for both measurements.

### 2.5. Biofilm Profiling Using Microsensors

The proposed microsensor was used for recording DO and pH profiles within an SOB biofilm. A single measurement provided instantaneous, 7-point DO and pH profiles in a biofilm section of 350 μm in thickness. Complete profiles were obtained by performing two consecutive measurements, placing the needle across the biofilm with the aid of a micromanipulator resulting in a 14-point profile acquired in 60 s. The novel microsensor performance for biofilm profiling was assessed using DO and pH profiles acquired using a commercial DO microsensor (OXI-25, Unisense, Aarhus, Denmark) and a commercial pH microsensor (PH-25, Unisense, Aarhus, Denmark). The identical positioning of microsensors through the biofilm was ensured using a micromanipulator. The profiles recorded using commercial microsensors were obtained moving the microsensors through the experimental system in 50 μm steps using the micromanipulator. The entire profile acquisition using commercial microsensors required 30 s for each monitoring point.

The biofilm used to validate the operation of the microsensor was grown in a biofilm lab-scale reactor. A flat plate bioreactor (FPB), described in [21], was seeded with 40 mL of a sludge of 3.5 g·VSS L^−1^ from a biotrickling filter for the biological removal of hydrogen sulfide [7]. A mineral medium solution (prepared according to [37]) was supplied to the reactor using Na_2_S_2_O_3_ as a substrate. The hydraulic residence time during the biofilm growth was set at 8 h, and the internal recycle flow was adjusted at a linear velocity over a biofilm of 10 m·h^−1^. Finally, the reactor was operated at temperature and pressure conditions under which the microsensor was characterized and calibrated in order to ensure its optimal performance during biofilm profiling.

## 3. Results and Discussion

### 3.1. IrOx Electrodeposition on Pt Electrodes

**Figure 2:**
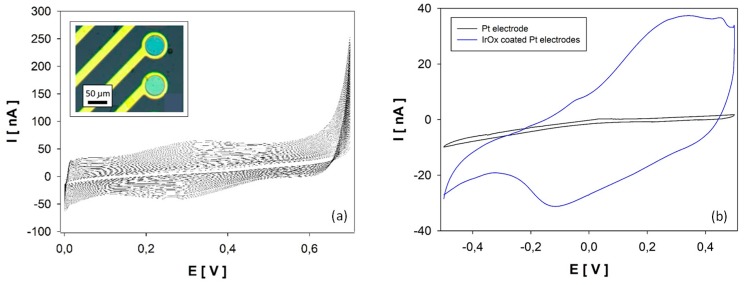
(**a**) Electrochemical IrOx synthesis results on a Pt 50 μm disk electrode. Inset: Image of two Pt electrodes coated with an IrOx film. (**b**) Cyclic voltamperometry results in PBS solution on a bare Pt electrode and an IrOx-coated electrode.

The Pt electrodes were coated with IrOx in order to perform potentiometric measurements for pH. Results obtained during the synthesis of IrOx film are shown in Figure 2a. Voltamperogram data revealed that, after each sweep potential, an IrOx layer was grown on the electrode, and, as a result, the measured current in the successive peaks increased. Current densities obtained during electrochemical IrOx film formation (25.46 A·m^−2^) showed similar and even higher values than these in similar works (12.25 A·m^−2^) [34]. These results confirmed the adequate application of anodic electrodeposition for IrOx synthesis in current Pt electrodes. Modified electrodes were optically examined after IrOx electrodeposition and showed their characteristic blue color, revealing that the procedure gave rise to a homogeneous IrOx coating over the entire electrode surface.

The morphological characterization of coated electrodes was completed by assessing electrode roughness from electrode capacity quantification. The electrode capacity, which is directly proportional to its surface, was measured by cyclic voltamperometry in a phosphate buffer solution (PBS) between −0.5 V and 0.5 V, with a scan rate of 10 mV·s^−1^. Results presented in Figure 2b show that electrode capacity increased from 1.43 to 30.96 nA·V after IrOx films electrodeposition. The increase of the modified electrode capacity and thus the electrodes’ effective surface confirmed the formation of an IrOx structure with a high roughness. Considering that the pH effect on electrode potential is caused by protons placed in the interstices of the IrOx structure [34], these results highlight the optimal characteristics of prepared electrodes for pH detection.

### 3.2. DO and pH Microsensor Response Characterization

**Figure 3:**
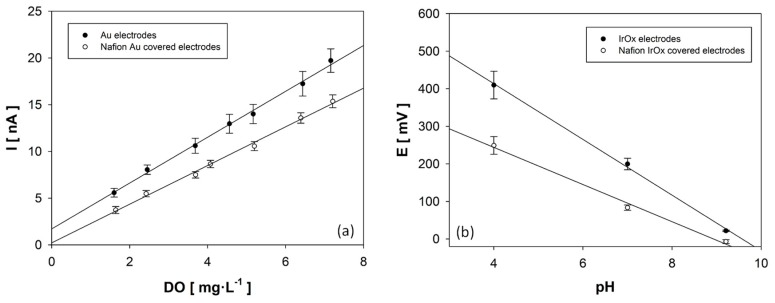
Calibration of the MEA sensor against DO (**a**), gold electrodes, and pH (**b**), IrOx electrodes. Calibrations were carried out before (●) and after (○) the deposition of the Nafion^®^ membrane (n = 7).

The electrochemical behavior of the novel microsensor was fully characterized in order to assess their performance for DO and pH monitoring. This procedure was also used to quantify the effect of a Nafion^®^ protective layer on the microsensor sensitivity. Both calibration curves were carried out before and after Nafion^®^ membrane deposition. Results obtained in the microsensor response during calibration are shown in Figure 3.

Calibration results obtained in naked needles displayed an excellent linearity (0.997) for oxygen detection between 0 and 8 mg·L^−1^ of DO. The experimental sensitivity was quantified at 2.06 ± 0.08 nA·mg^−1^·L, which is slightly lower than that of the 50 μm gold disk electrodes in the literature (2.41 nA·mg^−1^·L) [15]. Nevertheless, calibration results showed that the current increase between two consecutive DO concentrations was higher than the measurement error, which confirmed that the sensitivity of the developed microsensor is adequate for DO monitoring. The intercept of the oxygen calibration, although it was very close to zero, showed a residual current in the absence of oxygen due to the offset produced by hydrogen reduction on the electrode surface of approximately 1 nA. This value demonstrates the high selectivity of the gold electrodes for the detection of DO, as was observed in similar works [15,19,38]. The microsensor response against pH also exhibited a linear (0.999) super-Nerstian response between pH 4 and 9.21, with a sensitivity of −74.2 ± 0.7 mV·pH^−1^ comparable to the reported for IrOx pH sensors (between −63.5 and −77.6 mV·pH^−1^) [18,39,40]. Microelectrodes were modified after calibration by depositing Nafion^®^ over the needle. The deposition of several Nafion^®^ layers was assessed in terms of membrane thickness. Results revealed no differences on the thickness of the membrane depositing 1, 2, 3, or 4 Nafion^®^ layers, and 250 ± 25 nm membranes were obtained. The results obtained in DO and pH calibration after electrode modification are shown in Figure 3a,b, respectively. These results revealed that the linearity of the response was not affected by the presence of a Nafion^®^ film over electrodes, obtaining also correlation factors (r^2^) greater than 0.99. On the other hand, it was observed that the implementation of a protective membrane on the needle introduced a diffusion barrier for analytes, reducing microsensor sensitivity for DO and pH monitoring by 13.9% and 18.4%, respectively. Nevertheless, the protected needle had sensitivities of 1.73 ± 0.12 nA·mg^−1^·L and −60.5 ± 0.6 mV·pH^−1^ for DO and pH monitoring, respectively. In addition, calibration results also demonstrated a high reproducibility of the device for DO and pH detection as seen in the values of the error bars that were even lower after Nafion deposition. The characterization of microsensor response after the Nafion^®^ membrane deposition was completed by quantifying the detection (L_D_) and quantification (L_Q_) limits of the protected microsensor (Table 1). The value of L_D_ and L_Q_ for DO and pH detection shown in Table 1 are close to typical values reported for bare electrodes [15,18]. Thus, microsensor capacity for DO and pH monitoring was not significantly modified by Nafion^®^ protection.

### 3.3. Stability of Long-Term Microsensor Response

The performance of the Nafion^®^ membrane to avoid electrode fouling and passivation was assessed, monitoring microsensor sensitivity for DO and pH over 1000 h. The sensitivities were estimated from calibration data recorded periodically during the monitoring period. The modified microsensors were stored in a KCl (3 M) solution during the entire period in order to avoid Nafion^®^ dehydration and the deterioration of electrodeposited IrOx films. The sensitivities estimated over time were compared both with bare needle sensitivity and the initial sensitivity (0 h). Results are shown in Table 2.

Results obtained in long-term sensitivity assessment presented a different trend between Au and IrOx electrodes. Specifically, the DO monitoring sensitivity decreased from 1.73 ± 0.12 to 1.67 ± 0.17 nA·mg^−1^·L after 1000 h, corresponding to a reduction of 3%, while the pH monitoring ranged between −60.5 mV·pH^−1^ and −67.7 mV·pH^−1^ but presented an almost constant sensitivity along the monitoring period. Results revealed that membrane protection improved microsensor long-term performance compared with naked electrodes [15] where 40% of sensitivity loss was quantified after 1000 h of operation. On the other hand, the aging of gold electrodes introduced some dispersion for DO monitoring as seen in the values of sensitivity errors presented in Table 2. Nevertheless, the dispersion of DO sensitivity presented herein was smaller than that observed in the bare electrodes [15], highlighting that the implementation of a Nafion^®^ membrane for electrode protection successfully increased the measurement reproducibility.

The difference observed between the Au and IrOx electrode trends was provoked by the nature of the Nafion^®^ membrane. In this sense, the proton exchange membrane allows for the contact of the cations contained in the medium (mainly protons) with the surface of the electrodes. The oxidative nature of some of those species then passivated the gold electrode surface over time [41]. For this reason, Au electrode sensitivity for DO detection decreased over time, while IrOx-coated electrode sensitivity for pH detection remained constant.

These results validate the implementation of Nafion^®^ membrane protection to increase the applicability of the proposed microsensor for biofilm monitoring by increasing its response stability and enabling its use for performing long-term monitoring.

### 3.4. Validation of the Microsensor Operation

**Figure 4:**
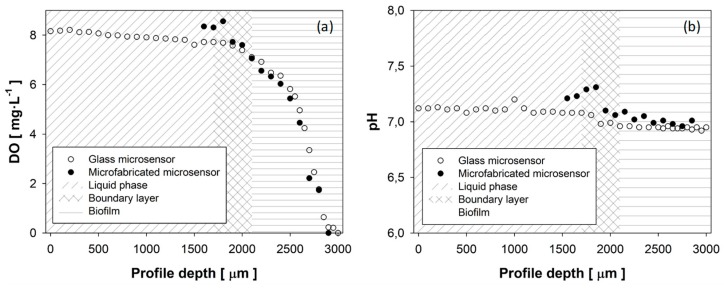
DO (**a**) and pH (**b**) profiles recorded with commercial microsensors and the microfabricated microsensor.

The multi-analyte microsensor performance for biofilm monitoring was validated, assessing its operation for the recording of DO and pH profiles within an SOB biofilm grown on a lab-scale biofilm reactor. The operation of the microsensor was validated comparing the DO and pH profiles with the ones obtained using a DO commercial microsensor (OXI-25, Unisense, Aarhus, Denmark) and a pH commercial microsensor (PH-25, Aarhus, Unisense, Denmark). The profiles recorded using both types of sensors were performed on the same biofilm point placed at 8 cm from the reactor inlet. The DO and pH profiles were obtained recording the sensor response from the gas–liquid interface to the deeper biofilm section. Microsensors were placed on a micromanipulator, and profiles were obtained as detailed in Section 2.5. Results obtained during novel microsensor validation are shown in Figure 4.

DO profiles using both microsensors through a reactor section (Figure 4a) were recorded from the gas–liquid interface (corresponding to a depth profile of 0 μm) to the deeper biofilm (corresponding to a profile depth of 3000 μm). Results revealed that the high spatial resolution achieved using the novel microsensor enables an accurate estimation of the thicknesses of the liquid film, the boundary layer, and the biofilm. According to [42], the flat section at the beginning of the recorded profile was identified as the completely mixed bulk phase, while the profile section with the sharpest DO gradient corresponded to the biofilm. The boundary layer was identified as the thickness between the liquid phase and the biofilm. Therefore, it was observed that, at the sampled point, the liquid film had a thickness of 1800 μm, while the thickness of the biofilm was only 700 μm. It was also identified that mass transfer resistance was located in a thin 200 μm layer between the two phases.

Comparing the measured DO concentration using a Clark-type microsensor and the novel microsensor, results denoted some differences, mainly in the liquid phase. In this sense, DO concentrations of 7.81 mg·L^−1^ and 8.31 mg·L^−1^ were measured near the liquid–biofilm interface using the Clark-type microsensor and the novel microsensor, respectively. The difference of 0.5 mg·L^−1^ measured by both microsensors can be attributed to the liquid phase dynamics along the reactor. Nevertheless, both DO profiles recorded within the biofilm are in good accordance throughout the entire biofilm section, showing how the DO concentration decreases from 7.77 to 6.55 mg·L^−1^ through the liquid–biofilm interface, and a complete oxygen consumption at a biofilm depth of 700 μm.

The pH profiles recorded using the Clark and multielectrode microsensor are shown in Figure 4b. The pH results presented some difference along the entire profile. In this sense, the biggest differences were observed in pH measurements within the liquid phase, where the pH measured using the multi-analyte microsensor was 0.25 pH units higher. These differences can be attributed both to the effect of the reactor dynamics on the pH of the liquid phase and to the equilibration time required by IrOx electrodes. The stabilization period is the consequence of the time required for the aqueous solution to fill the interstices of the IrOx structure, described in [18], which was between 1 and 2 min. Considering that entire profiles were recorded in two consecutive seven-point measurements, these results highlighted that the first measurement was performed before IrOx electrode stabilization was complete. For this reason, the first seven profile points exhibited greater differences against conventional microsensor measurements. The second measurement was recorded after the equilibration time and showed a high agreement with pH measurements performed with a conventional glass microsensor, where pH differences were below 0.1 pH units. These results revealed that the equilibration time was higher than that described in the literature [18,34]. The operating principle of conventional pH glass microsensors is based on proton permeability through the glass membrane, with a typical size exceeding 200 μm [8]; for this reason, the membrane size limits the final sensor dimensions. In this sense, the novel multi-analyte microsensor increased pH measurement resolution to 50 μm, improving the microsensor’s performance for pH biofilm profiling. The pH profile presented an almost flat gradient both within the liquid phase and within the biofilm. Small differences were observed between the pH measured inside the liquid phase, 7.05, and within the biofilm, 6.95. These results highlighted that the proton diffusion rate was high enough to flatten the pH gradient. Therefore, the pH profile through a reactor section was not affected by proton generation during the biological oxidative activity of hydrogen sulfide to sulfate [43].

The biofilm profiling results obtained using the multielectrode microsensor exhibited smaller differences against conventional microsensors than similar studies found in the literature [15]. The high concordance between DO and pH profiles recorded using the two types of microsensors showed that the substitution of conventional glass substrates, with a typical thickness above 500 μm [15,38], by a thin Kapton^®^ film (127 μm) reduced the invasive character of microsensor measurements, improving the performance of the microsensor. In this sense, it was possible to reduce the deviations in the measurements caused by the perturbations of the needle on the biofilm structure. High sensitivities together with minimally invasive characteristics exhibited by the novel microsensor enabled the obtainment of oxygen and pH microprofiles to define the mass transport boundary layer and to locate the boundary layer liquid side and the biofilm surface in a biofilm section. In this sense, assuming diffusive transport within the boundary layer, the boundary layer thickness has been typically used to further determine mass transfer coefficient [42,44].

Results also showed that the multi-electrode design enables the simultaneous recording of DO and pH profiles in a single measurement. Moreover, biofilm profiling using the novel microsensor simplified the experimental setup required and reduced the acquisition time of a complete DO and pH profile. Thus, the developed microsensor enables the continuous measuring of DO concentration and pH at different depths. This is, to the best of our knowledge, the first microdevice capable of performing these type of measurements. The comprehensive monitoring of both species through biofilms opens up a range of applications, such as the performance of respirometric and titrimetric studies [45] in biofilm systems to assess biomass activity, the characterization of mass transport phenomena, and biofilm dynamics modeling.

## 4. Conclusions

This work reports the development of a novel multi-analyte microsensor based on MEMS technologies for simultaneous monitoring of DO and pH. Several technological modifications were implemented in order to improve its performance for biofilm profiling. The change of conventional microfabrication substrates by a thin polymer of 127 microns clearly improved microsensor performance, reducing the damage caused by the needle insertion during biofilm profiling. The IrOx layer formed on Pt microelectrodes showed an excellent performance as pH sensor. Needle protection with a thin Nafion^®^ membrane avoided electrode fouling, providing long-term stability.

Results showed the capacity of microsensors to quantify concentration gradients of different species within biofilms. In addition, the possibility of integrating two microsensors, specially designed to obtain a seven-point profile in a single measurement, introduced in this paper for the first time, clearly simplifies the equipment and the procedure necessary to record concentration profiles within biofilms. Critical future prospects have been established. Future works must pursue Nafion^®^ membrane thickness decreases to increase sensor sensitivities as well as the integration of a robust and stable Ag/AgCl electrode as pRE.

## Figures and Tables

**Table 1:** L_D_ and L_Q_ for the measurement of DO and pH of the Nafion^®^ coated needle (n = 7).

	L_D_	L_Q_
DO monitoring [mg·L^−1^]	0.05 ± 0.01	0.17 ± 0.02
pH monitoring [pH units]	0.05	0.08

**Table 2:** DO and pH sensitivity of a Nafion^®^ coated needle over time in comparison with the uncoated bare ones. Times correspond to hours from the calibration of the bare needle just before modifying the sensor with the Nafion^®^ membrane.

		DO Detection Sensitivity [nA·mg^−1^·L]	pH Detection Sensitivity [mV·pH^−1^]
Bare Needle		2.06 ± 0.08	−74.2 ± 0.7
Coated Needle	0 h	1.73 ± 0.12	−60.5 ± 0.6
	150 h	1.78 ± 0.14	−67.7 ± 0.6
	300 h	1.62 ± 0.08	−62.6 ± 0.6
	850 h	1.64 ± 0.16	−63.5 ± 0.6
	1000 h	1.67 ± 0.17	−62.9 ± 0.6
